# The Wiedemann-Franz law in the putative one-dimensional metallic phase of PrBa_2_Cu_4_O_8_

**DOI:** 10.1038/srep03261

**Published:** 2013-11-20

**Authors:** A. F. Bangura, Xiaofeng Xu, N. Wakeham, N. Peng, S. Horii, N. E. Hussey

**Affiliations:** 1 H. H. Wills Physics Laboratory, University of Bristol, Tyndall Avenue, Bristol BS8 1TL, United Kingdom; 2 Magnetic Materials Laboratory, RIKEN, 2-1 Hirosawa, Wako, Saitama 351-0198, Japan; 3 Department of Physics, Hangzhou Normal University, Hangzhou 310036, China; 4 MPA-CMMS, Los Alamos National Laboratory, P.O. Box 1663, Los Alamos, NM 87545, USA; 5 Surrey Ion Beam Centre, ATI, Faculty of Engineering and Physical Sciences, University of Surrey, Guildford GU2 7XH, United Kingdom; 6 Graduate School of Energy Science, Kyoto University, Yoshida-Honmachi, Sakyo-ku, Kyoto 606-8501, Japan; 7 High Field Magnet Laboratory, Institute for Molecules and Materials, Radboud University 6525 ED Nijmegen, The Netherlands; 8 These authors contributed equally to this work.

## Abstract

The nature of the electronic state of a metal depends strongly on its dimensionality. In a system of isolated conducting chains, the Fermi-liquid (quasiparticle) description appropriate for higher dimensions is replaced by the so-called Tomonaga-Luttinger liquid picture characterized by collective excitations of spin and charge. Temperature is often regarded as a viable tuning parameter between states of different dimensionality, but what happens once thermal broadening becomes comparable to the interchain hopping energy remains an unresolved issue, one that is central to many organic and inorganic conductors. Here we use the ratio of the thermal to electrical conductivities to probe the nature of the electronic state in PrBa_2_Cu_4_O_8_ as a function of temperature. We find that despite the interchain transport becoming non-metallic, the charge carriers within the CuO chains appear to retain their quasiparticle nature. This implies that temperature alone cannot induce a crossover from Fermi-liquid to Tomonaga-Luttinger-liquid behaviour in quasi-one-dimensional metals.

Understanding how the electronic state evolves in quasi-one-dimensional (q1D) metals as coupling between individual chains is strengthened or weakened, and determining the energy scale for the Fermi-liquid to Tomonaga-Luttinger liquid (FL-TLL) crossover, remain profound theoretical problems that are relevant to a host of organic and inorganic q1D conductors^1^. Temperature *T* is often regarded as a viable tuning parameter between states of different dimensionality in q1D metals. For *k_B_T* < 2*t*_⊥_, the interchain hopping integral, charge hops coherently in all three dimensions, albeit with anisotropic velocities. Once thermal broadening is comparable to the warping of the Fermi sheets however, hopping between chains is predicted to become incoherent, leading to a putative 3D-1D dimensional crossover^2^ and contrasting behaviour in the intra- and inter-chain resistivities at high *T*. As a result, signatures of TLL physics are expected to emerge with increasing temperature^3^.

In an alternative picture, it is argued that interchain coherence in a q1D FL is robust provided the intrachain scattering rate Γ < *ε_F_*, the Fermi energy^4^. Accordingly, there is no dimensional crossover with increasing *T*, and by inference, no FL-TLL crossover at elevated temperatures - the crossover to non-metallic behaviour in the interchain resistivity being simply due to the emergence of a second, incoherent hopping process which shorts out the small, but nonetheless metallic component^4^.

In order to address this outstanding issue experimentally, it is necessary to identify both a material whose anisotropic resistivity exhibits behaviour consistent with predictions for a thermally-induced dimensional crossover and a physical property that shows marked differences in the putative TLL and FL regimes. According to both theory and experiment, the Wiedemann-Franz (WF) law is a viable litmus test of TLL physics in the bulk. The WF law states that the ratio of the thermal *κ* to the electrical conductivity *σ* at a given *T* is equal to a constant called the Lorenz number, *L*_0_ = (*π*^2^/3)(*k_B_*/*e*)^2^. For FL systems, only small (*O*(1)) deviations from the WF law are expected (with an *effective* Lorenz ratio *L* = *κ*/*σT* ≤ *L*_0_)^5^,^6^,^7^,^8^, reflecting the fact that heat and electrical currents, though relaxed differently by inelastic scattering, are carried by the same fermionic quasiparticles (Although the WF law is most applicable in the zero temperature (elastic scattering) limit, the law is found to hold equally well at finite temperatures whenever large-angle (inelastic) scattering processes dominate). For certain classes of non-FL metals, the WF law is also obeyed at low *T*, provided some or all of the fermionic carriers remain long-lived^9^. In a TLL however, the Lorenz ratio is predicted to be enhanced^10^, by orders of magnitude under certain commensurate conditions^11^, due to the idea that both elastic and inelastic scattering processes affect the flow of charge (carried by holons) more profoundly than the flow of entropy (carried by spinons). Recently, a marked enhancement of the Lorenz ratio was observed in the q1D purple bronze Li_0.9_Mo_6_O_17_^12^, that appeared to diverge with decreasing temperature, consistent with expectations for a TLL with repulsive interactions^10^.

The q1D cuprate PrBa_2_Cu_4_O_8_ (Pr124) contains weakly coupled 1/4-filled zigzag chains, oriented along the crystallographic *b*-axis, that give rise to electronic properties that are among the most anisotropic known in existence^13^,^14^. The interchain resistivities *ρ_a_* and *ρ_c_*, while metallic (and FL-like) at low *T*, become non-metallic above *T*_max_ ~ 150 K (i.e. they both decrease with increasing *T*)^13^. The intrachain resistivity *ρ_b_*, on the other hand, remains metallic at all finite *T*, suggesting a purely 1D metallic state at elevated temperatures. Moreover, both photoemission lineshapes (in Zn-doped Pr124)^15^ and the optical response (in pristine Pr124)^16^ contain features claimed to be consistent with TLL theory.

Here, we examine the evolution of the Lorenz ratio in Pr124, through a combination of irradiation-induced and substitutional disorder, and find that *L*/*L*_0_ ≤ 1 (to within our experimental accuracy) both below *T*_max_ and above, following a *T*-dependence similar to that obeyed in elemental metals. This correspondence indicates that despite its extreme electrical anisotropy, Pr124 appears to display conventional metallic behaviour for all *T* ≤ 300 K and that there is no thermally-induced FL-TLL crossover beyond *k_B_T* > 2*t*_⊥_ ~ 5 meV^13^,^14^. Comparison with Li_0.9_Mo_6_O_17_ and with theory suggests that it is possibly the non-local nature of the electron correlations, rather than the degree of electrical anisotropy, that leads to the manifestation of TLL physics in the latter.

## Results

**Figure 1:**
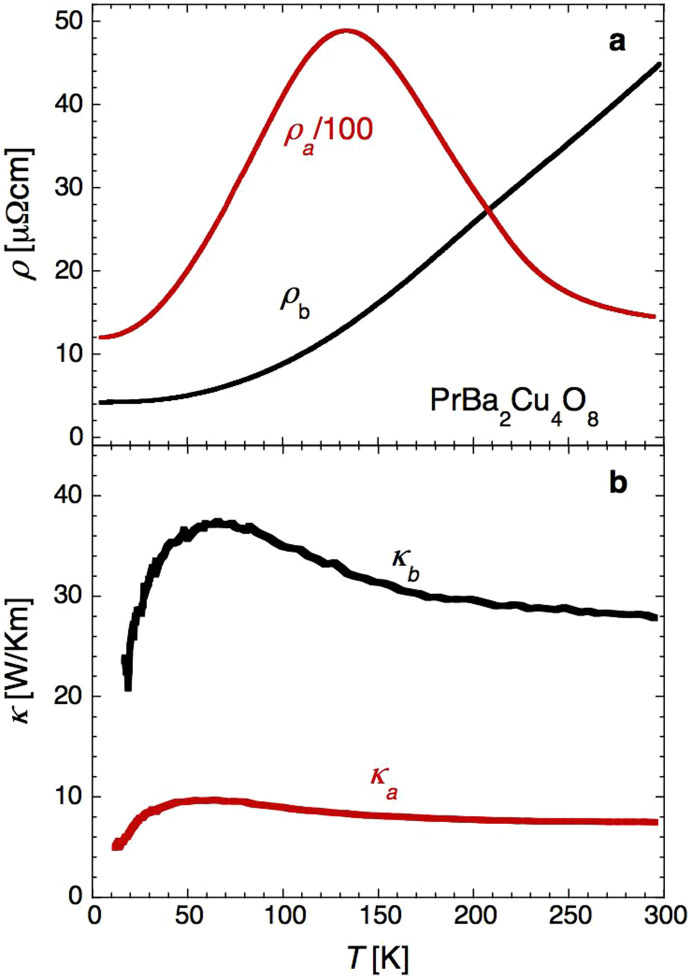
Electrical and thermal conductivities of quasi-1D PrBa_2_Cu_4_O_8_ (Pr124). (a). Intra- (*ρ_b_*) and inter-chain (*ρ_a_*) resistivity of Pr124 as a function of temperature. Note that *ρ_a_* has been scaled by a factor of 1/100. (b). Intra- (*κ_b_*) and inter-chain (*κ_a_*) thermal conductivity as a function of temperature.

The top panel in Figure 1 shows the electrical resistivity of stoichiometric Pr124 for current flow parallel (I//*b*) and perpendicular (I//*a*) to the conducting chains. Below 100 K, the electrical resistivity of Pr124 varies as *ρ* ~ *T*^2+*δ*^ (0 ≤ *δ* < 1) in all three crystallographic directions (data for I//*c* not shown)^13^, consistent with expectations for a q1D FL with dominant electron-electron scattering^17^. The corresponding resistivity anisotropy *ρ_a_*:*ρ_b_*:*ρ_c_* ~ 300:1:1000 at low *T*^18^. At higher temperatures, the *T*-dependence of *ρ_a_*(*T*) (and *ρ_c_*(*T*)) changes from metallic to non-metallic, while *ρ_b_*(*T*) remains metallic and monotonic. In this temperature regime, *ρ_b_*(*T*) becomes *T*-linear (see Fig. 1).

The corresponding thermal conductivity data for heat flow parallel and perpendicular to the chains is shown in the bottom panel of Figure 1. Like the electrical resistivity, it too displays a marked anisotropy, albeit reduced due to the additional phonon contribution *κ_ph_* to both *κ_a_* and *κ_b_*. Indeed, given the extreme resistive anisotropy, it is reasonable to assume that *κ_a_* is purely phononic in origin.

Thus, the *ab*-plane anisotropy in the thermal conductivity can be attributed either entirely to the electronic contribution *κ_e_* within the chains or to a combination of *κ_e_* and additional anisotropy in the phonon spectrum and/or phonon scattering rate. (Other contributions, e.g. due to spin fluctuations and/or magnons associated with the magnetic ordering of the copper ions in the CuO_2_ plane (*T*_N_ = 220 K) or of the Pr ions sandwiched between the CuO_2_ planes (*T*_N_ = 17 K)^19^, are expected to be isotropic within the *ab*-plane and therefore do not contribute to the anisotropy in *κ*). In isostructural YBa_2_Cu_4_O_8_ (Y124), *κ_ph_* scales only with sample dimensions at low *T*^20^, implying that the mean phonon velocity is also isotropic within the *ab*-plane. In SrCuO_2_, an insulating cuprate with an identical zigzag chain structure to Pr124, the *ab*-plane anisotropy in *κ_ph_* is small, of order 20% and only weakly *T*-dependent, between 0.5 K and 300 K^21^. In Pr124, the additional (electronically inert) CuO_2_ bilayers sandwiched between the chains will undoubtedly act to reduce the overall phonon anisotropy relative to that in SrCuO_2_. Hence it is reasonable to assume that the bulk of the difference *κ_b_* − *κ_a_* is due solely to heat flow of the charge carriers within the CuO chains. Before we discuss the effective *L*(*T*) estimated from *κ_b_* − *κ_a_* however, we first present evidence that near room temperature at least, *L* ~ *L*_0_ independent of any assumptions involving the anisotropy in *κ_ph_*.

**Figure 2:**
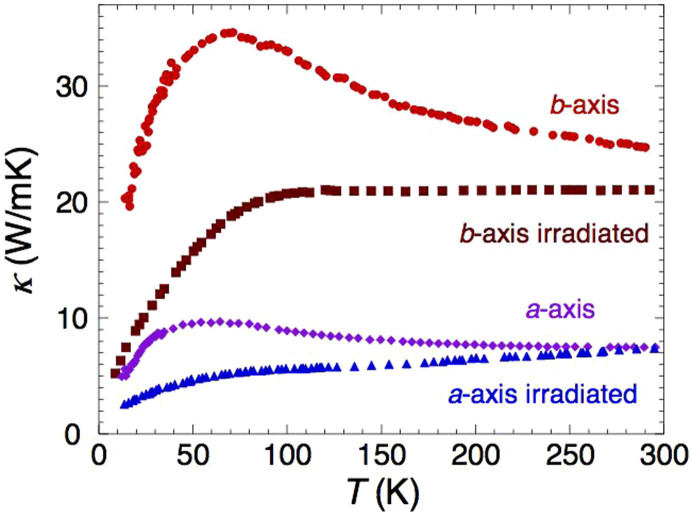
Thermal conductivity data on Pr124 single crystals before and after proton-irradiation. *κ_b_*(*T*) and *κ_a_*(*T*) data on Pr124 crystals before and after receiving the same radiation dose from a 4 MeV proton beam at 300 K for 12 hours. The thickness of the two samples was small compared to the penetration depth of the protons, ensuring homogeneous damage throughout the crystals. Note that *κ_a_*, the phonon contribution, at room temperature is insensitive to the level of disorder.

Figure 2 shows *κ_b_*(*T*) and *κ_a_*(*T*) data on Pr124 samples cut from the same large single crystal before and after receiving proton irradiation. The phonon peaks in the virgin crystals were both strongly suppressed, verifying that a substantial level of impurities was introduced by the radiation exposure. (At this level of irradiation, we expect the defects to be predominantly single oxygen vacancies). Significantly, *κ_a_* at 300 K was found to be insensitive to irradiation damage. (The room temperature value of *κ_a_* on a second non-irradiated crystal with a lower phonon peak, and therefore presumably higher disorder levels, was also coincident with those shown in Figure 2). This finding indicates that the dominant scattering mechanism for phonons at high *T* is phonon-phonon (Umklapp) scattering rather than phonon-impurity scattering. By contrast, *κ_b_* was found to be suppressed by ~20% upon irradiation. It is evident therefore that the change in *κ_b_* at room temperature arises solely from changes in *κ_e_*.

**Figure 3:**
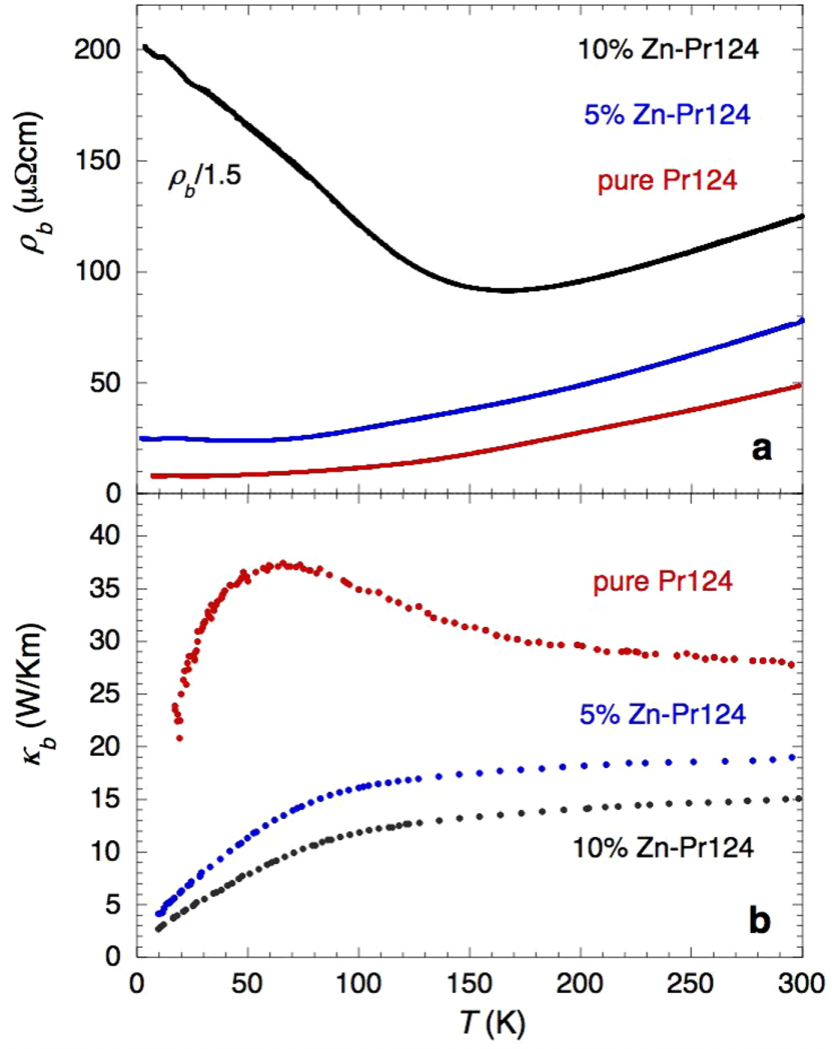
Effect of Zn substitution on the in-chain charge dynamics of Pr124. (a). Temperature dependence of the in-chain electrical resistivity of Pr124 single crystals with different levels of Zn substitution (chemical formula PrBa_2_(Cu_1−*x*_Zn*_x_*)_4_O_8_), as indicated. For clarity, *ρ_b_* of the 10% sample has been divided by a factor of 1.5. (b). Corresponding in-chain thermal conductivity data.

With this in mind, we now proceed to examine the effect of substitutional disorder on the room temperature Lorenz ratio of Pr124. (Measuring the electrical resistivity of the irradiated crystals was not an option since post-irradiation annealing of those electrical contacts that had degraded during the irradiation process would have caused a recombination of the majority of defects). Figure 3 shows *ρ_b_*(*T*) (top panel) and *κ_b_*(*T*) (bottom panel) measurements on pure, 5% and 10% Zn-doped Pr124 single crystals. Note the upturns in the *ρ_b_*(*T*) curves of the two Zn-doped crystals, consistent with a previous localization study of Pr124^22^. Note too the similarity in the *κ_b_*(*T*) curves for the irradiated and 5% Zn-doped samples (implying a similar defect density) and the fact that the small phonon peak seen in Fig. 1 is strongly suppressed upon Zn substitution. According to our simulations, the defect density of the irradiated crystals was estimated to be of order 0.1–0.2%. This is much less than the nominal Zn content in our Zn-doped crystals. It should be noted however that for low doping concentrations, Zn is believed to substitute Cu ions primarily on the CuO_2_ plane, rather than on the CuO chain.

**Figure 4:**
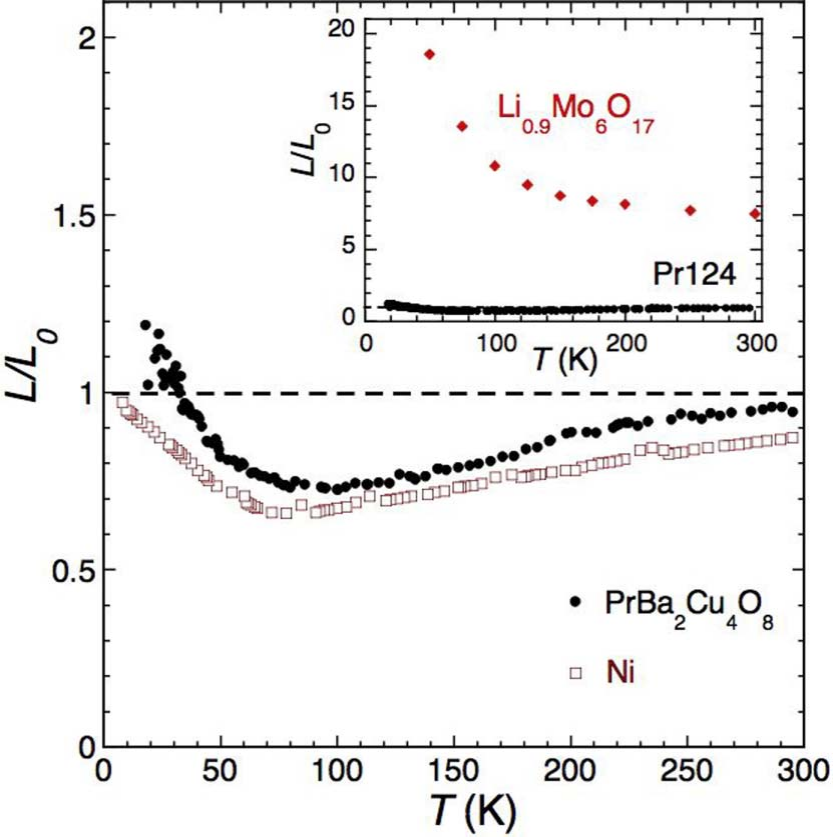
Verification of the Wiedemann-Franz law in Pr124. Solid circles: normalized WF ratio in Pr124. Open circles: corresponding *L*/*L*_0_ for Ni^23^. Inset: Comparison of *L*/*L*_0_ for Pr124 (solid circles) and Li_0.9_Mo_6_O_17_ (solid diamonds).

Assuming, as inferred from the irradiation experiments, that at high *T*, Δ*κ_b_* = Δ*κ_e_* (i.e. *κ_ph_* is insensitive to impurities), we can calculate Δ*κ_b_*/(Δ*σ_b_T*) for any combination of the three samples (where Δ*σ_b_* is the corresponding change in the electrical conductivity) and obtain an average value for the WF ratio of *L*/*L*_0_ = 1.15 ± 0.3 at *T* = 300 K. This value of *L*/*L*_0_ is entirely consistent, to within our experimental uncertainty, with the value derived for Zn-free Pr124 by assuming that the difference between *κ_a_* and *κ_b_* is wholly due to the contribution from the charge carriers within the chains (see Fig. 4 and subsequent discussion). This implies that the magnitude of the phonon anisotropy in Pr124 is of order or smaller than our experimental error, and is comparable to that found in SrCuO_2_^21^. More importantly, this analysis appears to confirm the preservation of the WF law in the putative ‘one-dimensional’ regime of Pr124, as we shall now discuss.

## Discussion

Figure 4 shows the resultant effective Lorenz ratio *L* ( = (*κ_b_* − *κ_a_*)/*σ_b_T*, where *σ_b_* = 1/*ρ_b_*) for the data shown in Fig. 1 normalized to the Lorenz number *L*_0_ ( = 2.45 × 10^−8^ V^2^K^−2^). For comparison, we also show in Fig. 4 the corresponding plot of *L*/*L*_0_ for Ni^23^. Similar behaviour is also seen in other elemental metals such as Cu^24^ and Co^23^. With decreasing temperature, *L*/*L*_0_ for Pr124 remains within 20% of its room temperature value and follows an almost identical *T*-dependence to that found in elemental Ni, dropping below unity at intermediate temperatures (presumably due to the different weighting of small- and large-angle scattering on the heat and charge currents) and recovering as *T* approaches the elastic scattering limit at *T* = 0. This pattern contrasts markedly with what is observed in the q1D purple bronze Li_0.9_Mo_6_O_17_^12^, reproduced in the inset to Fig. 4, for which the effective Lorenz ratio is found to be several times larger than *L*_0_ and to diverge with decreasing temperature^12^. It should be stressed here that according to theory^11^, the Lorenz ratio in a TLL is highly sensitive to both *d*, the deviation from commensurate filling and to *D*, the ratio of the elastic to el-el Umklapp scattering rates. However, as noted in Fig. 1 of Ref. 12, for values of *D* relevant to our crystals (0.1 < D < 1) and the temperature range of our experiments (10 K < *T* < 300 K), *L*/*L*_0_ in a TLL is always enhanced by a factor of 2 or higher, for *all* values of *d* considered.

Fig. 4 encapsulates the key result of this study, namely the equivalence of the *T*-dependence of *L*/*L*_0_ in Pr124 to that found in ordinary, elemental metals. (The observed excess of *L* over *L*_0_ below 30 K is attributed to experimental uncertainties, e.g. in our estimate of the distance between thermocouple contacts, or to any residual low-*T* anisotropy in *κ_ph_* that has been hitherto ignored). Given the striking violation in the WF law found in Li_0.9_Mo_6_O_17_^12^, the observation of WF law *verification* in Pr124 can be viewed as primary experimental evidence that the metallic state in Pr124 retains its quasiparticle (FL) nature for all *T* ≤ 300 K. This finding contrasts with the reported emergence of TLL behaviour at finite frequencies^16^ or energies^15^ in Pr124 and challenges the widely-held viewpoint^2^ that temperature alone can induce a radical change in the nature of the electronic state in q1D metals once *k_B_T* > 2*t*_⊥_. It also highlights a fundamental difference between probing physical processes at or near the Fermi level with increasing temperature and probing physical processes at finite frequencies or equivalently, at energies away from *ε_F_*.

It is not yet clear whether these findings support Gutman and Maslov's original argument that interchain transport remains coherent above *T*_max_^4^, since the loss of interchain coherence (i.e. once the scattering rate 1/*τ* < 2*t*_⊥_) may not necessarily induce a crossover to TLL physics^25^. Intriguingly, the strictly *T*-linear *ρ_b_*(*T*) observed in Pr124 above *T*_max_ is consistent both with expectations for a 1/4-filled TLL with repulsive interactions^2^ and, from simple phase-space arguments, for a q1D FL whose Fermi surface warping has been smeared out^17^.

Which factor or factors ultimately determine the contrasting electronic states in Pr124 and Li_0.9_Mo_6_O_17_ is an important open question. One obvious measure of the degree of three-dimensionality in a q1D FL is the interchain hopping integral *t*_⊥_ (For simplicity, we assume here that 2*t*_⊥_ is the same in both directions orthogonal to the conducting chains). As stated above, in Pr124, a consistent value of 2*t*_⊥_ ~ 5 meV has been obtained from a variety of studies^13^,^14^,^18^,^22^,^26^. For Li_0.9_Mo_6_O_17_, band structure calculations suggest that 2*t*_⊥_ ~ 36 meV^27^, while the resistive anisotropy (*ρ_a_*/*ρ_b_* ~ 0.5(*t*_//_/*t*_⊥_)^2^), combined with an estimate of the intrachain bandwidth *t*_//_ from angle-resolved photoemission^28^, gives 2*t*_⊥_ ~ 15 meV. Thus it would appear that the magnitude of *t*_⊥_ does not, by itself, determine the nature of the electronic state in q1D metals. According to Castellani and co-workers, for short-range interactions, the FL ground state is believed to be stable to *any* finite interchain coupling^29^. For sufficiently small *t*_⊥_ and sufficiently long-range interactions however, Kopietz *et al.* argue that the response of a system to experimental probes can be indistinguishable from that of a TLL, even in the dc limit, and characterized by the same TLL exponents that would exist if *t*_⊥_ = 0 30. This contrasting behaviour implies that it is the nature of the effective correlations, rather than the strength of the interchain coupling, that ultimately determines whether a metallic system near the 1D boundary displays FL or TLL phenomenology. In this regard, it is perhaps worth noting that repulsive interchain interactions are known to enhance pairing in the spin-triplet channel of q1D superconductors^31^,^32^,^33^, a scenario consistent with the recent observation of a strong violation of the Pauli paramagnetic limit in Li_0.9_Mo_6_O_17_^34^,^35^.

In summary, we have demonstrated that for 10 K ≤ *T* ≤ 300 K, the effective Lorenz ratio in Pr124 has a magnitude and *T*-dependence that is identical (to within our experimental uncertainty) with that found in elemental metals. Thus, despite its interchain resistivity having characteristics associated with a loss of dimensionality at elevated temperatures (i.e. above *T*_max_ ~ 150 K), the electronic state of Pr124 does not appear to undergo any transition or crossover to TLL physics once *k_B_T* > 2*t*_⊥_, in contrast to certain prevailing theoretical arguments. There is now a significant body of experimental evidence suggesting that the effective dimensionality of the conduction electrons in Pr124 can be modified through changes in temperature^13^, intrachain scattering^22^ and magnetic fields^14^,^26^ (i.e. once the energy scale of the relevant perturbation exceeds 2*t*_⊥_). However, it would appear that signatures of TLL physics are only manifest in spectroscopic measurements carried out at high frequencies^15^,^16^. This dichotomy is also seen in non-metallic magnetic systems on the 1D boundary^36^. It will be highly informative to see whether the electronic state of other q1D conductors, in particular Li_0.9_Mo_6_O_17_ and the organic Bechgaard salts (TMTSF)_2_*X* (*X* = ClO_4_, PF_6_) show a similar response to the different perturbations.

## Methods

Pr124 crystals were grown using a self-flux method in MgO crucibles in a high-pressure furnace (11 atm of oxygen)^37^. Bar-shaped samples (approximate dimensions 500 × 100 × 30 *μ*m^3^) were cut from a large rectangular as-grown sample with edges parallel to the crystallographic *a*- and *b*-axes. For the thermal conductivity measurements, we employed a modified steady-state method in which a temperature gradient, measured using a differential thermocouple, is set up across the sample through a pair of calibrated heat-links attached to each end^38^. The heat links determine the power entering and leaving the crystal, thus ensuring that any power loss due to radiative losses and heat conduction through the thermocouple wires to the heat-bath is known. Provided the difference between the power entering and leaving the sample is less than 20%, the power through the sample can be taken as the average of the input and output power. This condition was satisfied for all *T* ≤ 300 K and the total power loss typically fell to below 2% at *T* ~ 200 K.

In order to test the validity of the WF law, accurate measurements of the electrical resistivity are paramount. In a q1D conductor, extreme care is required to short out the sample electrically in the two directions orthogonal to the chain and thus ensure that current flow between the voltage contacts is uniaxial. This was achieved here by coating conductive paint across the entire sample in both directions perpendicular to the current flow. The zero-field measurements were carried out for 4.2 K < *T* < 300 K in a ^4^He dipping cryostat.

## Author Contributions

S.H. grew the single crystals and commented on the manuscript. N. P. performed the proton irradiation experiments. A.F.B., X.X., N.W. and N.E.H. designed and performed the transport experiments and co-wrote the paper.
